# Comparative Approach to Define Increased Regulatory T Cells in Different Cancer Subtypes by Combined Assessment of CD127 and FOXP3

**DOI:** 10.1155/2011/734036

**Published:** 2011-08-28

**Authors:** Marc Beyer, Sabine Classen, Elmar Endl, Matthias Kochanek, Martin R. Weihrauch, Svenja Debey-Pascher, Percy A. Knolle, Joachim L. Schultze

**Affiliations:** ^1^Laboratory for Genomics and Immunoregulation, LIMES-Institute, University of Bonn, Carl-Troll-Street 31, 53115 Bonn, Germany; ^2^Institute for Molecular Medicine and Experimental Immunology, University of Bonn,, 53105 Bonn, Germany; ^3^Clinic I for Internal Medicine, University of Cologne, Kerpenerstr. 62, 50924 Cologn, Germany

## Abstract

In recent years an increase of functional CD4^+^CD25^+^ regulatory T cells (T_reg_ cells) has been established for patients with solid tumors, acute leukemias, and lymphomas. We have reported an expanded pool of CD4^+^CD25^high^ T_reg_ cells in patients with chronic lymphatic leukemia (CLL), multiple myeloma (MM) as well as its premalignant precursor monoclonal gammopathy of undetermined significance (MGUS). In healthy individuals, low-level expression of CD127 on T cells in addition to the expression of FOXP3 has been associated with T_reg_ cells. Here, we demonstrate that the expanded FOXP3^+^ T-cell population in patients with colorectal cancer, CLL, MGUS, MM, follicular lymphoma, and Hodgkin's disease are exclusively CD127^low^ T_reg_ cells and were strongly suppressive. A significant portion of CD127^low^FOXP3^+^ T_reg_ cells expressed only low levels of CD25 suggesting that the previously reported expansion of CD25^+^ T_reg_ cells underestimates the true expansion. The assessment of CCR7 and CD45RA expression on the expanded CD4^+^CD127^low^FOXP3^+^ T_reg_ cells revealed an increase of both naïve as well as central and effector memory T_reg_ cells in peripheral blood. Our data strongly support superiority of combined CD127 and FOXP3 analysis in comparison to CD25 and FOXP3 assessment for further quantification of T_reg_ cells in malignant diseases.

## 1. Introduction

CD4^+^CD25^+^ regulatory T cells (T_reg_ cells) are expanded in murine tumor models, and their deletion can lead to complete tumor regression [1]. In humans, T_reg_ cells are mostly enriched in the CD4^+^CD25^high^ T-cell population [2]. We and others have reported increased frequencies of CD4^+^CD25^high^FOXP3^+^ T_reg_ cells in cancer patients [1, 3]. However, the expansion of T_reg_ cells based on the assessment of CD25 is likely to underestimate the true expansion since FOXP3^+^ T cells are also present in the CD25^−/low^ fraction [4, 5]. Furthermore, molecular and functional characterization of this population is hampered by the inability to separate CD25^+^ T_reg_ cells from activated effector T cells. Two recent studies, however, have shown that reciprocal expression of the IL7 receptor (CD127) on FOXP3^+^ T_reg_ cells is most likely a more specific way to quantify FOXP3^+^ T_reg_ cells [5, 6]. This has been adopted lately for the quantification of T_reg_ cells in solid tumors [7–10] and hematologic malignancies [11–13], with one of the reports establishing CD127 as an even superior marker for the identification of T_reg_ cells in cancer patients [9]. However, no systematic analysis has been undertaken to establish CD127 as a superior marker for T_reg_-cell enumeration in cancer patients, and only one initial report of malignant melanoma patients has addressed reciprocal expression of CD127 and FOXP3 on T_reg_ cells in cancer patients independently of CD25 [9]. It is, therefore, necessary to determine whether CD127 is also a better marker for enumerating FOXP3^+^ T_reg_ cells in cancer patients in general by comparing T_reg_ cells numbers in a larger number of different tumor subtypes. Besides the integration of CD25^low/−^ FOXP3-expressing T_reg_ cells, analysis of CD127 might, furthermore, clarify contradictory results concerning frequencies as well as prognostic value of T_reg_ cells in cancer patients [14–16]. 

Similarly, there is still debate whether human CD4^+^CD25^high^FOXP3^+^ T_reg_ solely belong to the memory T-cell compartment [17]. Valmori et al. were the first to identify a T_reg_-cell population with a naïve phenotype (CCR7^+^CD45RA^+^), which they termed natural naïve T_reg_ cells [18]. As expected, the frequency of these naïve T_reg_ cells was relatively low in healthy individuals [19]. More recently, Seddiki et al. have described the persistence of a population of naïve CD45RA^+^ T_reg_ cells in adult life [20], which was further characterized by resistance to CD95L-induced cell death [21]. Recent data further supports that a population of naïve T_reg_ cells exist in healthy individuals that exerts suppressive function [22]. So far, our own observations suggested an increased frequency of naïve CD4^+^CD25^high^FOXP3^+^ T_reg_ cells in MM and MGUS [23]. However, previous findings were restricted to the CD4^+^CD25^high^ subpopulation excluding a significant fraction of T_reg_ cells from analysis. With the emergence of CD127 as a new marker separating T_reg_ cells from conventional T cells, the question whether the expanded T_reg_ cells in cancer patients are mainly antigen-experienced memory cells or also naïve T_reg_ cells needs reevaluation.

Here, we present clear evidence that FOXP3^+^ T cells derived from patients with CLL, MGUS, MM, follicular lymphoma (FL), Hodgkin's disease (HD), and colorectal cancer (CRC) are lacking CD127. This newly defined fully functional CD4^+^CD127^low^FOXP3^+^ T_reg_-cell population is expanded in all tumor entities as well as the premalignant MGUS supporting the hypothesis of increased T_reg_ cells as a rather early event during tumor development. Moreover, we demonstrate a significant increase of naïve CD4^+^CD127^low^FOXP3^+^ T_reg_ cells in peripheral blood of cancer patients while we could not detect an increase in lymph node biopsies of lymphoma patients. Finally, these data strongly support the assessment of CD127 expression—instead of CD25—in combination with FOXP3 for a more precise enumeration of T_reg_ cells in malignant diseases while functional characterization still relies on the combination of CD127 and CD25.

## 2. Material and Methods

### 2.1. Patients and Clinical Parameters

Following approval by the institutional review board of the University of Cologne, peripheral blood from 10 healthy individuals, 7 MGUS, 10 MM, 10 CLL and 6 patients with CRC (2 time points at least 1 month apart) was obtained after informed consent. For the assessment of T_reg_ cell numbers in lymph node biopsies, lymph nodes from 7 healthy donors, 6 patients with HD, and 7 patients with FL were analyzed following approval by the institutional review board of the University of Cologne. Patients were either untreated or had not received cytoreductive treatment for a period of at least 1 month prior to investigation. Characteristics of the patients studied are summarized in Tables 1 and 2.

### 2.2. Antibodies and FACS Analysis

Phenotype of T cells was defined by flow cytometry using the following antibodies: CD45RA-PE-Cy5 (HI100), CD127-PE (hIL-7R-M21), CD4-APC-Cy7 (RPA-T4), CD25-PE-Cy7 (M-A251, all from Becton Dickinson), CCR7-FITC (150503, R&D) as well as the corresponding isotype control antibodies. Intracellular staining for FOXP3 was performed with FOXP3-APC (PCH101, eBioscience) according to the manufacturer's recommendations [23]. Samples were acquired on a FACS LSR II and analyzed with FlowJo software (TreeStar Inc). Frequencies of CD4^+^CD127^low^FOXP3^+^ T cells are shown as percent values of CD4^+^ T cells.

### 2.3. Isolation of CD4^+^CD127^low^CD25^+/low^ and CD4^+^CD127^+^CD25^−^ T Cells and Assessment of Inhibitory Function

To assess the suppressive activity of CD4^+^CD127^low^CD25^+/low^ T cells, a modified MLR was performed as previously described [23]. Briefly, CD4^+^CD127^low^CD25^+/low^ and CD4^+^CD127^+^CD25^−^ T cells were stained with CD4, CD25, and CD127 mAb and sorted on a FACSDiVa or FACS Aria III (both BD Biosciences) and incubated for 20 hours with 10 U/mL IL-2 (Proleukin) and 0.5 *μ*g/mL anti-CD3 (OKT3) in X-VIVO 15 (BioWhittakker) [24]. Subsequently, CD4^+^CD127^+^CD25^−^ T cells were stained with 5,6-Carboxyfluorescin-Diacetat-Succinimidyl-Ester (CFSE, Sigma-Aldrich) and stimulated in X-VIVO 15 supplemented with 10% fetal calf serum, 100 U/mL penicillin/streptomycin and 2 mM glutamine (Invitrogen) with magnetic beads (Dynal Biotech,) coated with 5% anti-CD3, 14% anti-CD28 (9.3), and 81% anti-MHC class I (W6/32) at a ratio of 3 : 1 (cells : beads). To assess inhibitory capacity of T_reg_ cells from cancer patients, autologous CD4^+^CD127^low^CD25^+/low^ T_reg_ cells were added at a 1 : 1 ratio to the culture, and the proliferation of CD4^+^CD127^+^CD25^−^ T cells was determined by assessing CFSE dilution after four days of culture as described previously [23].

### 2.4. RNA Preparation and Quantitative Real-Time PCR

For analysis of CD127 mRNA expression, CD4^+^CD25^−^ and CD4^+^CD25^high^ T cells from five healthy donors and five CLL patients were purified as previously described [24]. The described technique is optimized for the isolation of human CD4^+^CD25^high^ T cells with high purity [23, 24]. Cells were reanalyzed after sorting and routinely showed >95% purity. Subsequently, the cells were lysed in TRIzol reagent (Invitrogen). 50–100 ng RNA were reverse transcribed using the Transcriptor First Strand cDNA Synthesis Kit (Roche, Penzberg, Germany). Rt-PCR was performed with the LightCyclerTaqman master kit and Universal ProbeLibrary Assay on a Light Cycler 480 II. Analysis was performed using Light-Cycler3 and RelQuant software using a calibrator normalized relative quantification based on *β*-2 microglobulin (B2M) expression. Primers used: CD127 forward, 5′- AAAGTTTTAATGCACGATGTAGCTT-3′; CD127 reverse, 5′- TGTGCTGGATAAATTCACATGC-3′; Probe 72; B2M forward, 5′- TTCTGGCCTGGAGGCTAT-3′; B2M reverse, 5′TCAGGAAATTTGACTTTCCATTC-3′; Probe 42.

### 2.5. Statistical Analysis

Comparison between paired or unpaired groups was performed using the appropriate Student's *t*-test. A *P*-value < 0.05 was defined as statistically significant. Due to the explorative nature of this study, no multiplicity adjustment procedures were performed. All statistical analyses were performed using the SPSS statistical software package (SPSS 19.0, SPSS Inc.). 

## 3. Results

### 3.1. Downregulation of CD127 mRNA Expression in CD4^+^CD25^high^ T_reg_ Cells from CLL Patients

**Figure 1:**
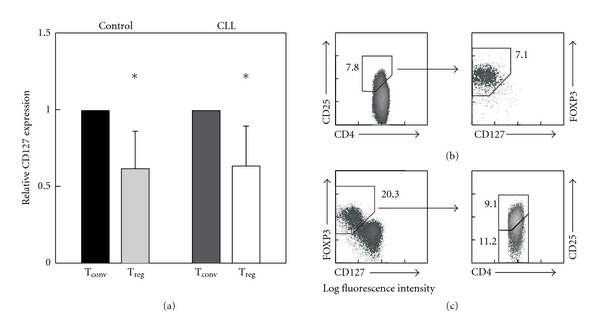
CD127 mRNA expression in CD4^+^CD25^high^ T_reg_ cells and integration of CD127 in the analysis of human T_reg_ cells. (a) Expression of CD127 mRNA in CD4^+^CD25^high^ T_reg_ cells and conventional CD4^+^CD25^−^ T cells in healthy donors (*n* = 5, control) and CLL patients (*n* = 5, CLL) as determined by qPCR (*, *P* < 0.05, Student's *t-*test). (b) Gating strategies for analysis of expression of CD127 in CD4^+^CD25^high^FOXP3^+^ T_reg_ cells or (c) CD25 expression in CD4^+^CD127^low^FOXP3^+^ T_reg_ cells.

As CD25 is not solely expressed on T_reg_ cells but also on activated conventional CD4^+^ T cells, and the downregulation of CD127 expression in CD4^+^CD25^high^ FOXP3^+^ T_reg_ cells from healthy donors has been reported [5, 6], we first assessed if CD127 downregulation is also apparent in CD4^+^CD25^high^ T_reg_ cells from cancer patients. We detected a significant downregulation of CD127 mRNA expression in CD4^+^CD25^high^ T cells from healthy donors (*n* = 5) as well as CLL patients (*n* = 5, *P* < 0.05, Figure 1(a)) by quantitative PCR indicating that CD127 expression might also be used to specifically identify CD4^+^FOXP3^+^ T_reg_ cells in cancer patients.

### 3.2. Coassessment of CD127 and FOXP3 for the Enumeration of Human T_reg_ Cells

Next the expression of CD127 in relation to FOXP3 and CD25 was evaluated by flow cytometry on CD4^+^ T cells. Gating on CD4 and CD25 with subsequent analysis of the CD4^+^CD25^high^ T_reg_-cell population for expression of FOXP3 and CD127 confirmed the downregulation of CD127 in CD4^+^CD25^high^FOXP3^+^ T_reg_ cells on protein level in healthy individuals (Figure 1(b)). However, assessing coexpression of CD127 and FOXP3 by CD4^+^ T cells without gating beforehand on the CD4^+^CD25^high^ T-cell population clearly revealed a significantly higher percentage of cells expressing FOXP3 but lacking CD127 (Figure 1(c)). Subsequent analysis of the CD127^low^FOXP3^+^ T_reg_-cell population for expression of CD25 demonstrated that gating on CD127 and FOXP3 identifies not only CD4^+^CD25^high^ T_reg_ cells but also T_reg_ cells expressing only low levels of CD25 (Figure 1(c)). The identification of this subpopulation of T_reg_ cells is of specific interest as up to now only T_reg_ cells expressing high amounts of CD25 were accessible to functional analysis.

### 3.3. Increase of CD4^+^CD127^low^FOXP3^+^ T_reg_ Cells in Cancer Patients

**Figure 2:**
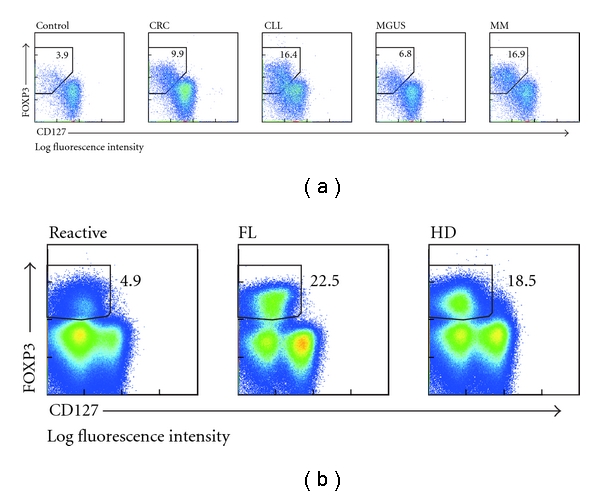
Frequency of CD4^+^CD127^low^FOXP3^+^ T_reg_ cells. Flow cytometric analysis of CD127 and FOXP3 expression in CD4^+^ T cells from (a) peripheral blood of a representative healthy individual (control) and representative patients with colorectal cancer (CRC), CLL, MGUS, and multiple myeloma (MM) and (b) lymph node biopsies from a healthy individual (reactive) and patients with follicular lymphoma (FL) and Hodgkin's diseases (HD).

**Figure 3:**
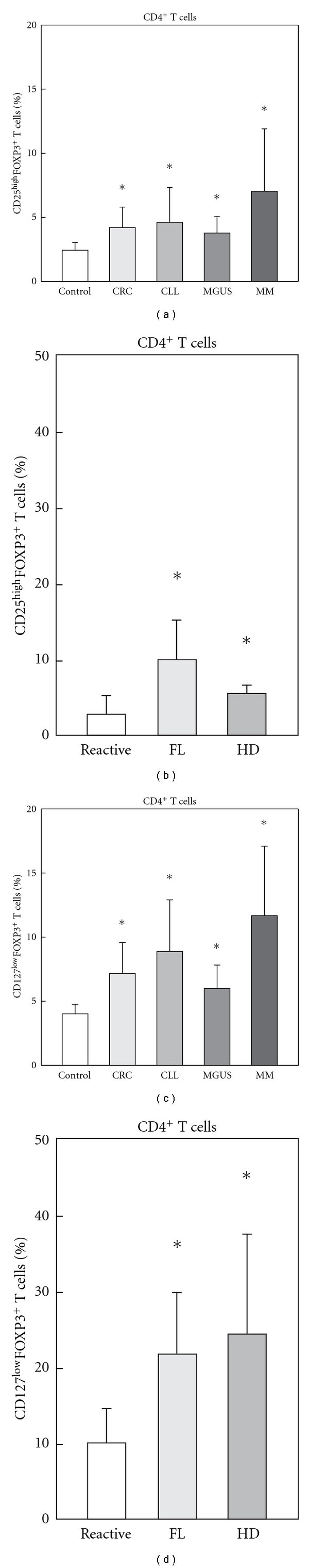
Assessment of T_reg_-cell frequencies. Frequency of CD4^+^CD25^high^FOXP3^+^ T_reg_ cells in (a) peripheral blood of 10 healthy donors (control), 12 colorectal cancer (CRC), 10 CLL, 7 MGUS, and 10 multiple myeloma (MM) patients and (b) 7 reactive lymph node biopsies from healthy individuals (reactive), 7 patients with follicular lymphoma (FL), and 6 patients with Hodgkin's disease (HD). (c) and (d) Frequencies of CD4^+^CD127^low^FOXP3^+^ T_reg_ cells in the respective groups. Error bars represent standard deviation (*, *P* < 0.05, Student's *t *test).

Inclusion of the CD25^low^ T_reg_-cell subpopulation in the enumeration of T_reg_ cells by defining human T_reg_ cells as CD4^+^CD127^low^FOXP3^+^ demands the reassessment of T_reg_-cell frequencies in cancer patients as the actual frequencies were probably underestimated until now. Comparison of healthy individuals with cancer patients revealed elevated levels of CD4^+^CD127^low^FOXP3^+^ T_reg_ cells in cancer and MGUS patients, as exemplified for individual patients in Figures 2(a) and 2(b). In total, frequencies of T_reg_ cells derived from peripheral blood of 12 patients with CRC, 10 CLL patients, 7 MGUS, and 10 MM patients as well as 10 healthy individuals were evaluated. In addition, lymph node biopsies from 7 patients with follicular lymphoma, 6 patients with Hodgkin's disease, and 7 reactive lymph nodes from healthy individuals were assessed for expanded T_reg_-cell numbers. Gating on CD4 and CD25 with subsequent gating on FOXP3 confirmed the already described increase of T_reg_ cells in patients with CRC, CLL, MGUS, MM, FL, and HD (Figures 3(a) and 3(b) and Tables 3 and 4). More important, when gating on FOXP3 and CD127 without using CD25 as primary inclusion criteria, frequencies of CD4^+^CD127^low^FOXP3^+^ T_reg_ cells in controls (4.1% ± 0.7%) were similar to previously published results (Figure 3(c) and Table 3) [2, 5, 6, 24]. In contrast, individuals with CRC (7.2% ± 2.4%, *P* < 0.005), CLL (8.9% ± 4.0%, *P* < 0.005), as well as MM (11.7% ± 5.4%, *P* < 0.005) showed significantly increased frequencies of CD127^low^FOXP3^+^ T_reg_ cells compared to healthy individuals (Figure 3(c) and Table 3). Even in MGUS patients, a significantly higher frequency of T_reg_ cells (6.0% ± 1.8%, *P* < 0.05) was observed (Figure 3(c) and Table 3), which is indicative of T_reg_-cell expansion as an early event in tumorigenesis. Similarly, we observed significantly increased frequencies of CD127^low^FOXP3^+^ T_reg_ cells in patients with FL (21.8% ± 8.0%, *P* < 0.01) and HD (24.4% ± 13.1%, *P* < 0.05) in comparison to reactive lymph node specimens from healthy individuals (10.1% ± 4.4, Figure 3(d) and Table 4). Moreover, the percentage of FOXP3^+^ cells within the CD4^+^CD127^low^ T-cell population was always higher than within the CD4^+^CD25^high^ population, suggesting that previous data only assessing a CD4^+^CD25^high^ phenotype have underestimated the absolute increase of FOXP3^+^ T_reg_ cells in cancer patients (Tables 3 and 4).

### 3.4. CD4^+^CD127^low^CD25^+/low^ T_reg_ Cells are Fully Functional in Cancer Patients

**Figure 4:**
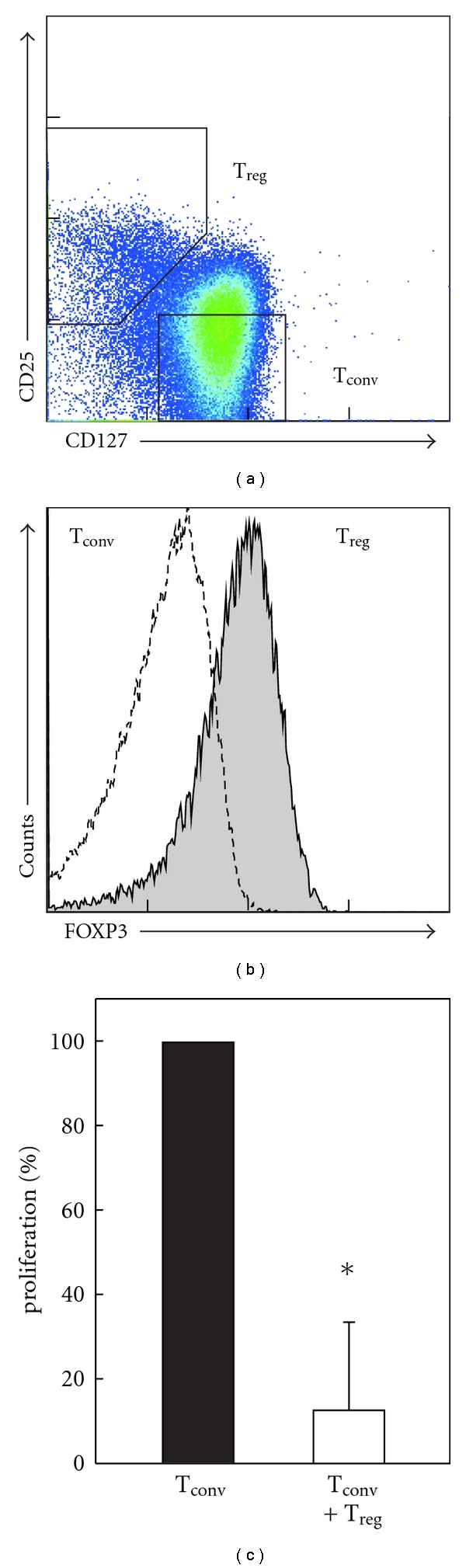
Functional analysis of CD4^+^CD127^low^CD25^+^ T_reg_ cells in cancer patients. (a) Sorting strategy for isolating CD4^+^CD127^low^CD25^+^ T_reg_ cells (T_reg_) as well as conventional CD4^+^CD127^+^CD25^−^ T cells (T_conv_). (b) Expression of FOXP3 in the corresponding T-cell populations. (c) Percentage of proliferation of CD4^+^CD25^−^CD127^+^ T_conv_ cells (black bar) alone or cultivated with CD4^+^CD127^low^CD25^+^ T_reg_ cells derived from CRC patients (*n* = 4) at a 1 : 1 ratio (white bar) both in the presence of CD3/CD28 mAb coated beads. Error bars represent standard deviation (*, *P* < 0.05, Student's *t *test).

As intracellular FOXP3 staining is not applicable for functional analysis of T_reg_ cells, classification of FOXP3^+^ T_reg_ cells based solely on cell surface markers is necessary. The characterization of FOXP3^+^ T_reg_ cells was best achieved when combining CD127 and CD25 (Figures 4(a) and 4(b)). We, therefore, used this combination of cell surface markers to sort T_reg_ cells for functional analysis. Staining for FOXP3 expression after sorting routinely showed purities of CD4^+^CD127^low^FOXP3^+^CD25^+/low^ T_reg_ cells >95 percent (Figure 4(b)). To determine whether the CD4^+^CD127^low^CD25^+/low^ T_reg_ cells from cancer patients are functional, we used an *in vitro *suppression assay. When activated with CD3/CD28 beads conventional CD4^+^CD127^+^CD25^−^ T cells, but not CD4^+^CD127^low^CD25^+/low^ T_reg_ cells, proliferate strongly. In the presence of CD4^+^CD127^low^CD25^+/low^ T_reg_ cells, this proliferation is suppressed (Figure 4(c)). These data clearly demonstrate that CD4^+^CD127^low^CD25^+/low^ T cells are FOXP3^+^ and that these cells are fully functional in CRC patients.

### 3.5. Naïve CD4^+^CD127^low^FOXP3^+^ T_reg_ Cells are Increased in Peripheral Blood of Cancer Patients

**Figure 5:**
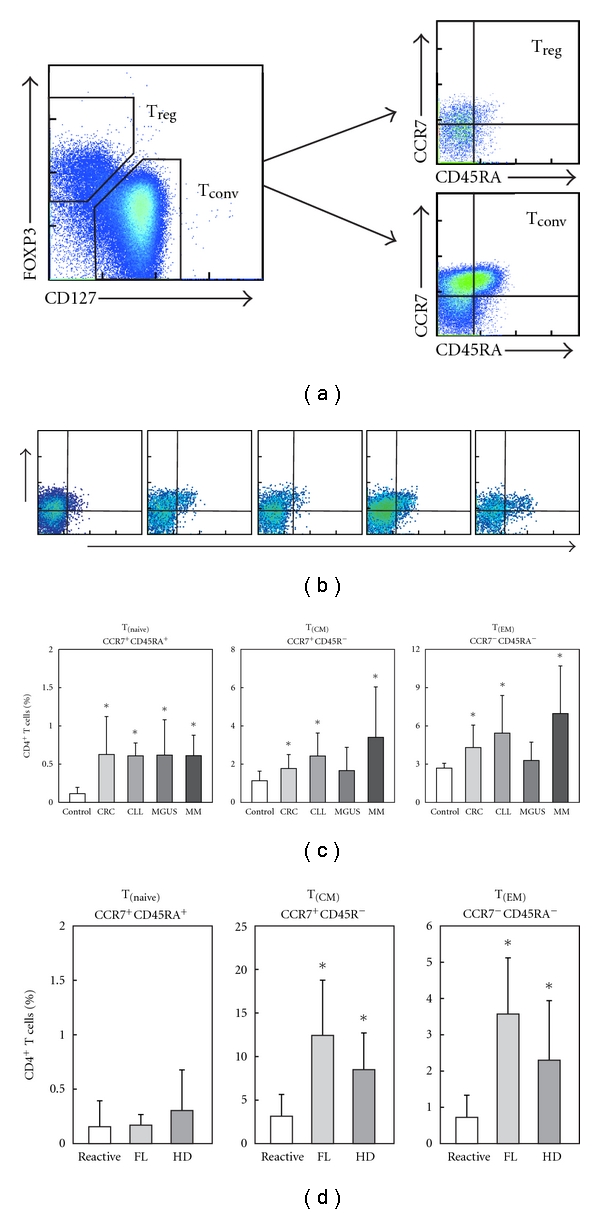
Assessment of naïve CCR7^+^CD45RA^+^CD4^+^CD127^low^FOXP3^+^ T_reg_ cells. (a) Frequencies of CCR7^+^CD45RA^+^ naïve CD4^+^CD127^low^FOXP3^+^ T_reg_ cells (T_naive_), CCR7^+^CD45RA^−^CD4^+^CD127^low^FOXP3^+^ central memory T_reg_ cells (T_CM_), and CCR7^−^CD45RA^−^CD4^+^CD127^low^FOXP3^+^ effector memory T_reg_ cells (T_EM_) were assessed in peripheral blood using gating on CD127 and FOXP3 with successive gating on CCR7 and CD45RA. (b) Flow cytometric analysis of naïve, central memory, and effector memory CD4^+^CD127^low^FOXP3^+^ T_reg_ cells in peripheral blood from a representative healthy individual (control) as well as representative patients with colorectal cancer (CRC), CLL, MGUS, and multiple myeloma (MM). Frequencies of regulatory T_naive_, T_CM_, and T_EM_ cells were assessed in (c) peripheral blood of CRC (CRC, *n* = 12), CLL (CLL, *n* = 10), MGUS (MGUS, *n* = 7), MM (MM, *n* = 10), and healthy individuals (control, *n* = 10) and (d) 7 reactive lymph node biopsies from healthy individuals (reactive), 7 patients with follicular lymphoma (FL), and 6 patients with Hodgkin's disease (HD). Error bars represent standard deviation (*, *P* < 0.05, Student's *t-*test).

In healthy individuals, T_reg_ cells have been shown to exist at all differentiation states, namely, naïve, central, and effector memory T_reg_ cells [18, 20, 25]. To determine which T_reg_-cell subpopulation is responsible for the increase of CD4^+^CD127^low^FOXP3^+^ T_reg_ cells in cancer patients, we determined the frequency of naïve, central, and effector memory cells within the T_reg_-cell compartment from healthy individuals, CRC, CLL, MGUS, and MM patients (Figure 5(a)) and compared these data with those previously described for CD4^+^CD25^high^ T_reg_ cells in healthy individuals as well as MGUS and MM patients [18, 23]. In healthy individuals, naïve CCR7^+^CD45RA^+^CD4^+^CD127^low^FOXP3^+^ T_reg_ cells were hardly detectable (Figures 5(b) and 5(c)). T_reg_ cells were almost exclusively of memory phenotype (Figures 5(b) and 5(c)). In contrast, in peripheral blood of CRC, CLL, and MM patients, a significant expansion of CD4^+^CD127^low^FOXP3^+^ T_reg_ cells with a naïve phenotype was observed (Figures 5(b) and 5(c)). The expansion of naïve T_reg_ cells was apparent as part of the T_reg_-cell pool as well as in relation to the total number of CD4^+^ T cells in cancer patients. This increase in naïve T_reg_ cells was further accompanied by an expansion of T_reg_ cells with a central as well as effector memory phenotype in all patient groups (Figures 5(b) and 5(c)). Interestingly, the observed expansion of naïve CD4^+^CD127^low^FOXP3^+^ T_reg_ cells was also detectable in MGUS patients (Figures 5(b) and 5(c)) further underlining that frequencies of naïve T_reg_ cells increase rather early during tumor development and progression. When assessing subpopulations of T_reg_ cells in lymph node specimens, we observed a predominance of CD4^+^CD127^low^FOXP3^+^ T_reg_ cells with a central-memory phenotype, with a significantly expanded population of central-memory T_reg_ cells apparent in patients with FL and HD (Figure 5(d)). In addition, we could also detect an increase in effector-memory T_reg_ cells (Figure 5(d)) while the pool of naïve T_reg_ cells was basically absent independent if reactive or diseased lymph nodes were analyzed (Figure 5(d)).

## 4. Discussion

Expansion of CD4^+^CD25^high^ T_reg_ cells within the tumor microenvironment and peripheral blood has so far been accepted as a hallmark of cancer [1, 26, 27]. Moreover, augmented T_reg_-cell frequencies have been linked to tumor stage, prognosis, and survival [1, 26, 27]. We present new evidence that the increase of T_reg_ cells in cancer was even underestimated previously due to suboptimal classification of T_reg_ cells. Integrating analysis of FOXP3 with the cell-surface molecule CD127 clearly demonstrates that significantly higher numbers of CD127^low^FOXP3^+^ T_reg_ cells are expanded in cancer patients in general. The assessment of CD127 instead of CD25 is clearly superior in enumerating T_reg_ cells in the diseased state.

Natural T_reg_ cells have been described as CD4^+^CD25^+^ T cells in mice [28], and initial reports in cancer patients relied solely on the assessment of CD4 and CD25 expression for the identification of T_reg_ cells [3, 29]. Only since the identification of the transcription factor FOXP3 lineage-specific marker of T_reg_ cells a more specific characterization of T_reg_ cells is possible [28]. In murine models, FOXP3 expression is strongly associated with the CD25^+^ T_reg_-cell population. However, even the inclusion of FOXP3 assessment has been interpreted differentially when assessing frequencies of T_reg_ cells in healthy individuals and cancer patients [23, 30]. The analysis of T_reg_ cells in humans has been further complicated as several studies reported FOXP3^+^ cells within the CD4^+^CD25^low^ or even CD4^+^CD25^−^ population [5], and even the reprogramming of T_reg_ cells into effector T cells has been reported [31]. Therefore, a more specific definition of T_reg_ cells based on unique or additional T_reg_-cell marker molecules is urgently needed. The introduction of CD127 as a new marker to distinguish T_reg_ cells from conventional T cells is an important improvement and will help to clarify several previous conflicting results in human T_reg_-cell biology, particularly in cancer patients. 

Several recent studies have adopted the approach to use CD127, CD25, and FOXP3 for the quantification of T_reg_ cells in tumor-bearing individuals and could demonstrate increased numbers of CD4^+^CD25^high^CD127^low^ T_reg_ cells in patients with solid tumors [7–10] and hematologic malignancies [11–13]. However, the majority of these reports focused solely on the enumeration of the T_reg_-cell compartment while at the same time focusing on only one tumor subtype. Only one study assessed T_reg_-cell numbers in more than one tumor subtype showing similar numbers of T_reg_ cells for all gastrointestinal tumor subtypes analyzed [8]. Furthermore, these studies did not systematically compare possible marker combinations to establish the most suitable approach to identify T_reg_ cells. This was analyzed in more detail in only one of the reports with the combination of CD127 and FOXP3 being the most appropriate combination to identify T_reg_ cells in patients with malignant melanoma [9].

The integration of CD127 permits to redefine the importance of CD25 expression on human T_reg_ cells. Up to now, high expression of CD25 allowed for an enrichment of CD4^+^ T cells with regulatory properties [2]. However, it is undisputed that neither all human T_reg_ cells are included by this approach nor that activated T cells expressing CD25 are excluded. Zelenay et al. could demonstrate a population of CD4^+^CD25^−^FOXP3^+^ T cells which can upregulate CD25 upon the depletion of all CD25 expressing cells and are able to replace the original T_reg_-cell population [4]. These data were a first hint that the expression of CD25 on T_reg_ cells is similarly regulated like its expression on conventional T cells [4]. Human T_reg_ cells need IL-2 for their survival and proliferation, and expression of the IL2R-*α* chain is certainly a prerequisite for IL-2 to exert its biological function [32]. However, the expression of CD25 is not homogenous and might also be dependent on the activation status and other exogenous factors [33]. 

Using CD127 and FOXP3 to define human T_reg_ cells demonstrates varying expression of CD25 in the CD4^+^CD127^low^FOXP3^+^ T_reg_-cell population. Additionally, the newly defined T_reg_-cell population comprises of significantly more T_reg_ cells compared to the traditionally defined CD4^+^CD25^high^ T_reg_ cells as demonstrated recently for malignant melanoma [9]. Coassessment of CD127 and FOXP3 to determine T_reg_ cells also resolves the uncertainty to differentiate between activated conventional T cells and T_reg_ cells in patients with active disease. This is of particular importance when only using CD4 and CD25 for the identification of T_reg_ cells in cancer patients, as contamination with effector T cells most frequently occurs when solely these two markers are used for analysis. As functional assessment of the CD4^+^CD127^low^FOXP3^+^ T_reg_-cell population is not possible as FOXP3 cannot be used for live studies of human T_reg_ cells, using expression of CD4, CD25, and CD127 is the best possible approximation. T cells isolated by this approach almost exclusively express FOXP3. Moreover, when isolated from cancer patients, this T_reg_-cell population exerts strong inhibition. 

Using a comparative approach analyzing different tumor subtypes from hematologic as well as epithelial origin, we demonstrate that all independent cancer patient groups studied uniformly show an expanded pool of CD4^+^CD127^low^FOXP3^+^ T_reg_ cells. We therefore postulate that expansion of T_reg_ cells is a general phenomenon in cancer patients. Moreover, since MGUS patients already have increased frequencies of T_reg_ cells, it is very likely that expansion of T_reg_ cells is an early event in the development of human tumors. Elevated T_reg_-cell levels might be associated with the progression from premalignant lesions that are still under control of the immune system to the uninhibited growth of malignant tumors.

The findings that naïve T_reg_ cells are increased both in the premalignant state as well as in cancer patients might further support this hypothesis. T_reg_ cells were first identified as antigen-experienced memory cells expressing CD45RO [2]. Only recently the existence of naïve T_reg_ cells in human adults has been reported [18, 20, 22, 23, 34], and the naïve T_reg_-cell population can be expanded *in vitro* while retaining its suppressive function [35, 36]. However, the physiological function of the naïve T_reg_-cell population remains unclear. Definition of T_reg_ cells as CD4^+^CD127^low^FOXP3^+^ has enabled us to verify the increase of naïve T_reg_ cells in MGUS and MM patients [23] and to extend these findings to CLL and CRC. 

The identification of an expanded pool of naïve T_reg_ cells in cancer patients opens new avenues to better understand the role of T_reg_ cells in malignant disease. Memory T_reg_ cells apparently cannot undergo self-renewal [37]. Therefore, the replenishment of an increased memory T_reg_-cell pool by differentiation of naïve T_reg_ cells into memory T_reg_ cells might be an alternative to the recently proposed conversion of conventional memory T cells to T_reg_ cells [37]. In fact, the increased pool of naïve T_reg_ cells with an unaltered frequency of memory T_reg_ cells in premalignant MGUS suggests that expansion of naïve T_reg_ cells is indeed preceding the expansion of memory T_reg_ cells following differentiation during tumor development. Besides the expansion of naïve T_reg_ cells through enhanced self-renewal and differentiation, other mechanisms have been proposed amongst them the interaction of CCR4 on T_reg_ cells with CCL22 released in the tumor microenvironment [38] as well as the conversion of conventional CD4^+^CD25^−^ T cells to T_reg_ cells trough TGF-*β* [39] or prostaglandin E_2_ [40]. How these factors influence the expansion of naïve T_reg_ cells needs further clarification and might in the end result in better strategies to target expanded T_reg_ cells in tumor patients. 

In conclusion this study demonstrates that CD4^+^CD127^low^FOXP3^+^ T_reg_ cells are increased in cancer patients. Definition of T_reg_ cells by combining CD127 and FOXP3 has the advantage of including not only T_reg_ cells expressing high levels of CD25 but also T_reg_ cells with low CD25 expression and excluding at the same time activated conventional T cells. Furthermore, the naïve T_reg_-cell population is expanded in all tumor bearing individuals.

## Figures and Tables

**Table:** (a) MGUS (peripheral blood)

ID	Gender	Age (yr)	Stage	Therapy	Paraprotein	T_reg_ (%)
1	M	37	MGUS	no tx	IgG/*λ*	3.6
2	F	84	MGUS	no tx	IgA/*κ*	7.0
3	F	86	MGUS	no tx	IgG/*κ*	7.5
4	F	62	MGUS	no tx	IgA	6.0
5	M	52	MGUS	no tx	n.a.	6.8
6	F	79	MGUS	no tx	n.a.	2.4
7	M	65	MGUS	no tx	IgM/*κ*	8.0

**Table:** (b) Multiple myeloma (peripheral blood)

ID	Gender	Age (yr)	Stage	Therapy	Paraprotein	T_reg_ (%)
8	M	62	MM I A	M	IgG/*κ*	16.5
9	F	39	MM I A	VAD, CAD, M, BMT	IgG/*κ*	7.4
10	M	59	MM II A	no tx	IgG/*κ*	10.2
11	F	74	MM I A	no tx	IgG/*λ*	4.5
12	M	86	MM I A	no tx	IgG/*κ*	6.7
13	M	60	MM II A	VAD, CAD, M, BMT	IgG/*κ*	16.9
14	F	52	MM II A	VID, C	IgG/*κ*	4.4
15	M	59	MM II A	TAD, CAD, M, BMT	IgG/*κ*	8.6
16	F	67	MM II A	VAD, CAD, M, BMT	IgG/*κ*	22.2
17	M	53	MM II A	no tx	IgG/*λ*	8.0

**Table:** (c) Chronic lymphocytic leukemia (peripheral blood)

ID	Gender	Age (yr)	Stage	Therapy	T_reg_ (%)
18	m	72	CLL A	no tx	6.1
19	m	62	CLL A	no tx	5.7
20	f	73	CLL A	no tx	5.6
21	m	60	CLL B	no tx	9.8
22	f	55	CLL B	no tx	10.6
23	m	73	CLL B	no tx	16.4
24	m	64	CLL B	no tx	7.4
25	m	39	CLL C	no tx	15.3
26	m	60	CLL C	no tx	6.2
27	m	54	CLL C	no tx	6.4

**Table:** (d) Colorectal cancer (peripheral blood)

ID	Gender	Age (yr)	Stage	Primary tumor	Sites of metastases	T_reg_ (%)
28	f	43	D	Rectum	Liver, bone, pararectal, para-aortal lymph nodes	7.5/10.4
29	f	32	D	Colon	Liver, spleen, ovaries, pelvis, peritoneum	5.7/9.3
30	m	57	D	Rectum	Lung	5.7/9.9
31	m	66	D	Colon	Liver	5.0/6.2
32	m	75	D	Colon	Liver	9.0/4.8
33	f	44	D	Colon	Liver	9.9/3.3

**Table:** (e) Healthy donors (peripheral blood)

ID	Gender	Age (yr)	T_reg_ (%)
34	m	66	5.4
35	m	67	6.6
36	m	55	3.9
37	m	50	6.5
38	m	47	4.5
39	m	46	3.5
40	m	46	4.8
41	m	62	4.1
42	m	45	2.6
43	f	44	4.0

Patient characteristics including gender, age at analysis, Durie and Salmon, Binet or Dukes stage, first diagnosis, primary tumor, sites of metastases, therapy, paraprotein, and frequency of T_reg_ cells. (f: female, m: male; therapy: A: Doxorubicin, BMT: autologous bone-marrow transplantation, C: Cyclophosphamide, I: Idarubicin, M: Melphalan, V: Vincristine, D: Prednisone, T: Thalidomide, no tx: no therapy, n.a.: not accessible.)

**Table:** (a) Follicular lymphoma (lymph node)

ID	Gender	Age (yr)	Stage	Therapy	T_reg_ (%)
44	m	59	FL I	no tx	23.9
45	m	46	FL I	no tx	13.4
46	f	58	FL I	no tx	26.8
47	m	73	FL II	no tx	19.6
48	f	66	FL II	no tx	20.0
49	m	59	FL II	no tx	13.2
50	m	57	FL II	no tx	22.5
51	m	65	FL II	no tx	37.5

**Table:** (b) Hodgkin's disease (lymph node)

ID	Gender	Age (yr)	Entity	Therapy	T_reg_ (%)
52	f	53	HD (ns)	no tx	37.8
53	m	44	HD (ns)	no tx	16.8
54	m	51	HD (ns)	no tx	43.9
55	m	19	HD (ns)	no tx	18.5
56	m	34	HD (ns)	no tx	16.9
57	f	25	HD (mc)	no tx	12.4

**Table:** (c) Healthy donors (reactive lymph nodes)

ID	Gender	Age (yr)	T_reg_ (%)
58	m	35	4.9
59	f	18	11.7
60	f	17	4.9
61	m	22	9.7
62	f	45	16.7
63	m	39	8.9
64	m	24	14.0

Patient characteristics including gender, age at analysis, first diagnosis, therapy, and frequency of T_reg_ cells. (f: female, m: male; no tx: no therapy; mc: mixed cellularity; ns: nodular sclerosing).

**Table 3:** Assessment of T_reg_-cell frequencies in peripheral blood.

	Control	Colon		CLL		MGUS		MM	
	Mean (SD)	Mean (SD)	*P*	Mean (SD)	*P*	Mean (SD)	*P*	Mean (SD)	*P*
CD4^+^CD127^low^	4.6 (1.3)	7.6 (1.5)	<0.001	8.3 (2.5)	<0.005	5.4 (1.3)	n.s.	11.2 (5.9)	<0.01
CD4^+^CD127^low^FOXP3^+^	**4.1 (0.7)**	**7.2 (2.4)**	**<0.005**	**8.9 (4.0)**	**<0.005**	**6.0 (1.8)**	**<0.05**	**11.7 (5.4)**	**<0.005**
CD4^+^CD127^low^FOXP3^+^CD25^high^	2.5 (0.6)	4.3 (1.6)	<0.005	4.7 (2.7)	<0.05	3.9 (1.3)	<0.05	7.1 (4.9)	<0.05
CD4^+^CD25^high^	2.8 (0.9)	7.6 (1.2)	<0.001	6.4 (1.8)	<0.001	4.5 (1.1)	<0.05	9.0 (5.3)	<0.01
CD4^+^CD25^high^CD127^low^	2.9 (0.9)	4.5 (1.2)	<0.005	4.5 (2.0)	<0.05	3.5 (1.3)	n.s.	7.0 (5.1)	<0.05
CD4^+^CD25^high^FOXP3^+^	2.1 (0.8)	4.2 (1.2)	<0.001	3.6 (1.7)	<0.05	2.5 (0.6)	n.s.	6.3 (4.5)	<0.05
CD4^+^FOXP3^+^	2.8 (0.9)	4.7 (2.1)	<0.05	4.7 (2.4)	<0.05	3.6 (1.1)	n.s.	7.7 (5.1)	<0.05

Definition of subpopulations based on expression of CD25, CD127, and FOXP3 (SD: standard deviation, n.s.: not significant).

**Table 4:** Assessment of T_reg_-cell frequencies in lymph node biopsies.

	Control	FL		HD	
	Mean (SD)	Mean (SD)	*P*	Mean (SD)	*P*
CD4^+^CD127^low^	54.7 (23.1)	68.6 (15.8)	>0.05	68.0 (12.7)	>0.05
CD4^+^CD127^low^FOXP3^+^	**10.1 (4.4)**	**21.8 (8.0)**	**<0.01**	**24.4 (13.1)**	**<0.05**
CD4^+^CD127^low^FOXP3^+^CD25^high^	3.1 (1.9)	11.7 (5.5)	<0.005	6.4 (2.3)	<0.05
CD4^+^CD25^high^	5.1 (2.9)	13.8 (7.1)	<0.05	11.2 (3.3)	<0.005
CD4^+^CD25^high^CD127^low^	4.1 (3.0)	16.3 (7.1)	<0.005	11.3 (5.2)	<0.05
CD4^+^CD25^high^FOXP3^+^	2.9 (2.6)	10.0 (5.4)	<0.01	5.6 (1.2)	<0.05
CD4^+^FOXP3^+^	10.3 (5.5)	19.4 (8.5)	<0.05	23.4 (12.0)	<0.05

Definition of subpopulations based on expression of CD25, CD127, and FOXP3 (SD: standard deviation, n.s.: not significant).

## Abbreviations

CLL: Chronic lymphatic leukemia;
MM: Multiple myeloma
MGUS: Monoclonal gammopathy of undetermined significance
CRC: Colorectal cancer
FL: Follicular lymphoma
HD: Hodgkin's disease
FOXP3: Forkhead box protein 3
T_reg_ cells: Regulatory T cells.
